# Partial Genome Characterization of Novel Parapoxvirus in Horse, Finland

**DOI:** 10.3201/eid2909.230049

**Published:** 2023-09

**Authors:** Jenni Virtanen, Maria Hautaniemi, Lara Dutra, Ilya Plyusnin, Katja Hautala, Teemu Smura, Olli Vapalahti, Tarja Sironen, Ravi Kant, Paula M. Kinnunen

**Affiliations:** University of Helsinki, Helsinki, Finland (J. Virtanen, L. Dutra, I. Plyusnin, K. Hautala, T. Smura, O. Vapalahti, T. Sironen, R. Kant, P.M. Kinnunen);; Finnish Food Authority, Helsinki (M. Hautaniemi);; Medical University of Gdansk, Gdynia, Poland (R. Kant)

**Keywords:** parapoxvirus, viruses, zoonoses, taxonomic classification, high-throughput nucleotide sequencing, horses, Finland

## Abstract

We report a sequencing protocol and 121-kb poxvirus sequence from a clinical sample from a horse in Finland with dermatitis. Based on phylogenetic analyses, the virus is a novel parapoxvirus associated with a recent epidemic; previous data suggest zoonotic potential. Increased awareness of this virus and specific diagnostic protocols are needed.

Parapoxviruses (PPVs) usually cause contagious skin infections in ruminants and occasionally infect other species such as humans (*1*). The genus *Parapoxvirus* encompasses the following recognized species: Orf virus, bovine papular stomatitis virus, pseudocowpoxvirus, red deerpox virus, and grey sealpox virus (GSEPV) (*2*). All of those, except GSEPV and red deerpox virus, are zoonotic. PPV genomes are usually 130–140 kb (*2*). Recently, poxviruses have emerged in humans and horses (*3*,*4*).

A severe infection caused by a parapox-like virus (F14.1158H) was first verified from a horse euthanized in Finland in 2013 (*5*). According to the short sequences (1.1 kb in total) obtained from envelope phospholipase (open reading frame [ORF] 011) and RNA polymerase subunit RPO147 (ORF056) genes, F14.1158H is most closely related to PPVs and is similar to the 585-bp sequences detected in lesions from humans after contact with horses and donkeys in the United States (*5*,*6*). However, the actual classification remained unclear because of limited sequence data and lack of amplification in numerous PPV PCR assays (*5*). No other clinical cases were confirmed until 2022, when an epidemic of dermatitis emerged in horses across Finland. PPV infection was subsequently identified in several cases using pan-PPV PCR (*7*) and Sanger sequencing (Appendix). Partial ORF011 sequences were 97% identical to the sequences from the 2013 case, with identity of 79%–87% to other PPVs (Appendix Table). This finding highlighted the need to properly characterize F14.1158H.

To better characterize the virus, we analyzed DNA extracted directly from a skin lesion of the 2013 equine case (*5*) and subjected it to next-generation sequencing with 2 different protocols (Appendix). The first protocol, relying on a pool of poxvirus primers, was insufficient to acquire enough sequence data. With a PCR-free approach, using enrichment of the viral DNA, we acquired as much as 121 kb of nucleotide sequence, almost the full genome, with coverage values of ≈100 in 5 contigs (BioProject no. PRJNA922554; GenBank accession nos. OQ248663–7). We noted the overall guanine-cytosine content to be 68.4%, which is similar to that of PPVs (*2*). We were unable to fully assemble and orient the data because we had no reference genome, a critical component in future investigations like ours. The lack of high-quality DNA and unsuccessful virus isolation attempts (Appendix) further complicated the sequencing process. In another study, researchers used a combination of short- and long-read sequencing to recover the full genome of the GSEPV (*8*). However, with our clinical sample, the small amount of DNA available for sequencing led to an alternative approach.

We conducted phylogenetic analysis for the following poxvirus core genes (*9*) (ORF numbers designated according to PPV ORFs) (*10*): DNA polymerase (ORF025), early transcription factor (ORF083), DNA-directed RNA polymerase subunit RPO132 (ORF101), and DNA topoisomerase type 1 (ORF062) (Figure). Consistent with earlier observations based on partial sequences of ORF11 and ORF056, F14.1158H grouped clearly closer to PPVs than other poxviruses (Figure, panel A), although distinctly separate from the 5 recognized species (Figure, panels B–D). We found the amino acid sequences to be more similar to PPV species than to other chordopoxviruses. For example, amino acid identity of DNA polymerase was 46%–60% between F14.1158H and viruses from other genera, 76%–80% between F14.1158H and PPVs, and 84%–95% among the previously recognized PPV species (Appendix Table 3). Within the PPVs, F14.1158H generally showed the second lowest pairwise nucleotide identity of the group (after the most divergent GSEPV) (Table); identities to other PPVs were 74%–83% (ORF025), 73%–83% (ORF083), 78%–87% (ORF101), and 84%–91% (ORF062). GSEPV was consistently furthest from F14.1158H, whereas F14.1158H identities to other PPVs were similar. A relatively high difference explains why F14.1158H was not detected by several PCRs designed for detecting PPV, which should be considered when designing diagnostic protocols. These phylogenetic results and sequence identities, together with the high guanine and cytosine content and disease characteristics, indicate that F14.1158H represents a novel PPV, designated equine parapoxvirus (EqPPV). The final taxonomic position and the possible differences of human and equine-derived variants (*6*) will require more data.

**Figure:**
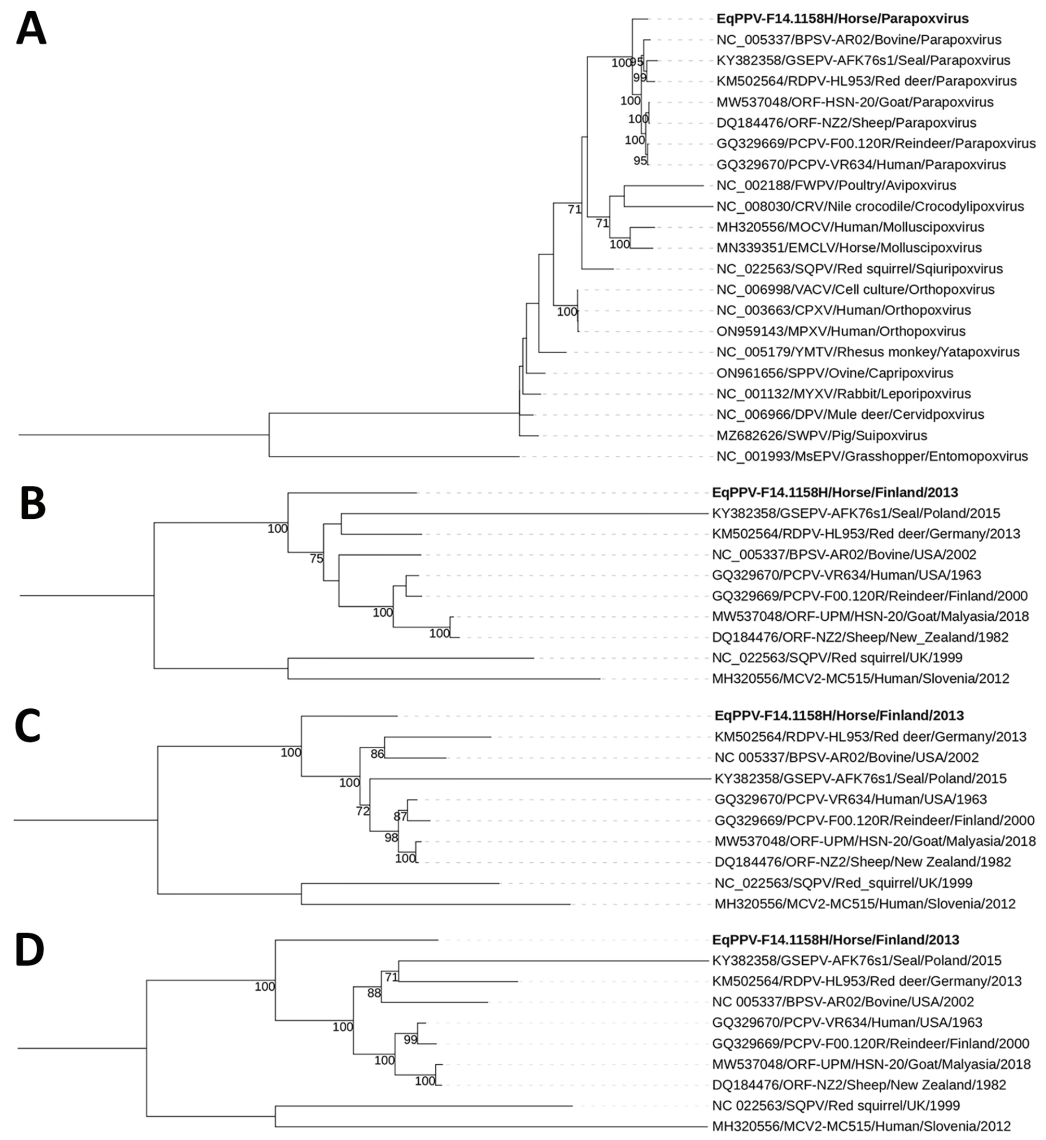
Phylogenies of PPV isolate F14.1158H from a skin lesion of an infected horse in Finland, 2013. A) Grouping of F14.1158H among all the genera of the subfamily Chordopoxvirinae in a phylogenetic tree based on amino acid sequences of the DNA polymerase (ORF025) gene. B–D) Grouping of F141158H among the genus *Parapoxvirus* in phylogenetic trees based on the nucleotide sequences of the early transcription factor (ORF083) (B), RNA polymerase (ORF101) (C), and topoisomerase 1 (ORF062) (D) genes. Bootstrap values >70% are shown next to the nodes. GenBank accession numbers are provided for reference sequences. MsEPV is used as an outgroup in panel A and SQPV and MOCV in panels B–D. Findings indicate that F14.1158H represents a novel PPV, designated EqPPV. CPXV, cowpox virus; CRV, crocodilepox virus; DPV, deerpox virus; EMCLV, equine molluscum contagiosum-like virus; EqPPV, equine PPV; FWPV, fowlpox virus; MOCV, molluscum contagiosum virus; MPXV, monkeypox virus; MsEPV, melanoplus sanguinipes entomopoxvirus; MYXV, myxoma virus; ORF, open reading frame; PPV, parapoxvirus; SPPV, sheeppox virus; SQPV, squirrelpox virus; SWPV, swinepox virus; VACV, vaccinia virus; YMTV, yaba monkey tumor virus.

**Table:** Nucleotide identity comparison between the PPV isolate F14.1158H and other PPVs and *Molluscum contagiosum* virus in 4 selected core genes based on DNA extracted directly from a skin lesion of an infected horse in Finland, 2013*

Core gene and virus	% Identity							
	*Molluscum contagiosum* (MC515)	Grey sealpox	Red deerpox	Bovine papular stomatitis (AR02)	Orf (UPM/HSN-20)	Orf (NZ2)	Pseudo-cowpox (F00.120R)	Pseudo-cowpox (VR634)
DNA polymerase (ORF025)								
Grey sealpox	62	NA	NA	NA	NA	NA	NA	NA
Red deerpox	68	79	NA	NA	NA	NA	NA	NA
Bovine papular stomatitis (AR02)	68	79	86	NA	NA	NA	NA	NA
Orf (UPM/HSN-20)	69	79	87	87	NA	NA	NA	NA
Orf (NZ2)	69	79	87	87	99	NA	NA	NA
Pseudocowpox (F00.120R)	69	79	87	87	94	94	NA	NA
Pseudocowpox (VR634)	70	80	88	88	95	95	98	NA
F14.1558H	69	74	81	81	82	82	82	83
Early transcription factor (ORF083)								
Grey sealpox	65	NA	NA	NA	NA	NA	NA	NA
Red deerpox	73	80	NA	NA	NA	NA	NA	NA
Bovine papular stomatitis (AR02)	74	80	90	NA	NA	NA	NA	NA
Orf (UPM/HSN-20)	73	79	88	89	NA	NA	NA	NA
Orf (NZ2)	73	79	88	89	99	NA	NA	NA
Pseudocowpox (F00.120R)	74	80	90	91	95	95	NA	NA
Pseudocowpox (VR634)	74	80	90	91	95	95	98	NA
F14.1558H	74	78	87	87	86	86	87	87
RNA polymerase (ORF101)								
Grey sealpox	73	NA	NA	NA	NA	NA	NA	NA
Red deerpox	79	84	NA	NA	NA	NA	NA	NA
Bovine papular stomatitis (AR02)	80	85	93	NA	NA	NA	NA	NA
Orf (UPM/HSN-20)	80	86	93	93	NA	NA	NA	NA
Orf (NZ2)	80	86	93	93	100	NA	NA	NA
Pseudocowpox (F00.120R)	80	86	92	94	97	97	NA	NA
Pseudocowpox (VR634)	80	86	93	94	98	98	98	NA
F14.1558H	80	84	89	91	91	91	91	91
Topoisomerase 1 (ORF062)								
Grey sealpox	62	NA	NA	NA	NA	NA	NA	NA
Red deerpox	68	79	NA	NA	NA	NA	NA	NA
Bovine papular stomatitis (AR02)	68	79	86	NA	NA	NA	NA	NA
Orf (UPM/HSN-20)	69	79	87	87	NA	NA	NA	NA
Orf (NZ2)	69	79	87	87	99	NA	NA	NA
Pseudocowpox (F00.120R)	69	79	87	87	94	94	NA	NA
Pseudocowpox (VR634)	70	80	88	88	95	95	98	NA
F14.1558H	69	74	81	81	82	82	82	83

*Findings indicate that F14.1158H represents a novel PPV, designated equine PPV. NA, not applicable; ORF, open reading frame; PPV, parapoxvirus.

Most known PPVs are zoonotic, and any novel virus detected in animals should be treated with concern (*6*). Thus, considering the tendency of PPVs to cause diseases in humans, EqPPV has a zoonotic potential. It is therefore important to sample humans and other animals in contact with infected horses. It is also critical to establish diagnostic protocols due to low specificity and sensitivity of pan-PPV PCR for EqPPV (Appendix). In terms of veterinary importance, this virus poses a threat for horses that could translate to financial losses for owners. The information provided here will inform development of proper diagnostic tools and also enable establishment of prevention measures.

Appendix Additional information for partial genome characterization of novel parapoxvirus in a horse, Finland.
