# Supplementary material for: Partial Genome Characterization of Novel Parapoxvirus in Horse, Finland
Source: Emerg Infect Dis. 2023 Sep;29(9):1941–4. doi: 10.3201/eid2909.230049 (PMC10461679; doi:10.3201/eid2909.230049)
Supplement: Appendix — Additional information for partial genome characterization of novel parapoxvirus in a horse, Finland. [file 23-0049-Techapp-s1.pdf]

Article DOI: https://doi.org/10.3201/eid2909.230049

EID cannot ensure accessibility for supplementary materials supplied by authors. Readers who have difficulty accessing supplementary content should contact the authors for assistance.

Partial Genome Characterization of Novel Parapoxvirus in Horse, Finland

Appendix

Testing of the samples from the 2021–2022 outbreak

Skin lesion swab samples from 25 horses having vesicular or other kind of acute dermatitis in the pastern area were collected by veterinarians at ten stables in Finland between December 2021 and March 2022. Given the history of circulation of PPV in horses in Finland *(1),* the samples were tested for the presence of the equine PPV. Swabs were incubated in 500 µl of Dulbecco's phosphate buffered saline + 0.2% bovine serum albumin in +4°C o/n and DNA was extracted using Viral RNA mini kit (Qiagen) according to manufacturer's instructions. Samples were then tested with pan-PPV-PCR *(2).* PCR products were run in 1.5% agarose gel. Products of correct size were purified with PCR purification kit or Gel extraction kit (GeneJET) and sequenced with Sanger sequencing.

PCR products from a total of 16 horses were sequenced, and poxvirus DNA was confirmed from nine (56%) of these. The rest were either host genome or had too many overlapping sequences to enable the confirmation of the presence or absence of poxvirus sequences. Sanger sequences from five horses from three stables had good enough quality for further analyses. These five sequences were compared with the ones obtained from the 2013 case (PPV variant F14.1158H; *1*) and selected strains from GenBank with phylogenetic analyses and nucleotide identity calculations (Appendix Figure and Appendix Table 1, methods described in 'PCR-free sequencing and sequence analysis' -section). Sequences of 2022 outbreak were 99%–100% identical to each other based on partial envelope phospholipase gene (ORF011) and 97% identical to the sequence of the 2013 case. They were only 79%–87% identical to other PPVs and were hence concluded to most likely represent the same PPV species, which infected the

index case in 2013. Sequences were deposited in GenBank with accession numbers OR112284-OR112288.

The pan-PPV-PCR showed poor specificity to equine PPV in horse skin samples. Furthermore, due to the sequence difference of EqPPV with the other PPVs (Appendix Table 1), we cannot trust the sensitivity either. Thus, the remaining 16 samples, from which we did not obtain clear PCR results or sequences, can't be considered negative. The obvious unspecificity results at least partially from host DNA in PCR products. It is important to develop more reliable diagnostic protocols to prepare for further epidemics.

Sequencing using a primer pool

DNA (a total of 4 µg at a concentration of 215 ng/µl) had been extracted from a skin lesion of an infected horse in our earlier study (*1*). In the first attempt to obtain enough sequence data for proper characterization from a very limited amount of sample, we used an enrichment PCR with a combination of poxvirus primers and next generation sequencing (NGS). PCR reactions contained 23.5 µl of water, 2 µl of the sample, 10 µl Taq buffer $(NH_4)_2SO_4$, 1 µl of 10 mM dNTP, 4 µl of 25 mM $MgCl_2$, 0.5 µl of Taq DNA polymerase (Thermo Scientific), and 1 µl of primer pool including 10 µM of each primer. The program was as follows: 95°C for 1 min, and 30 rounds of denaturation (95°C 30 s), annealing (50°C 30 s) and extension (72°C 4 min), followed by final extension (72°C 10 min). Two PCR reactions with different primer combinations were run: one using the combination of primers listed in Appendix Table 2 and another with reverse complementary sequences of the same primers. The aim was to amplify both the sequences flanked by the primers and the sequences outside the designed product by using the reverse complement. PCR products were combined and purified with GeneJET PCR purification kit (Thermo Scientific).

Libraries were prepared with Nextera XT DNA Sample preparation and Nextera XT Index kits (Illumina). Sequencing was performed with MiSeq (Illumina) and MiSeq reagent kit V2. For analyses, the sequence reads mapping equine genome were removed and the remaining reads were assembled with de novo assembly (MIRA 5 software *(3)*). Because the data contained very little poxvirus sequence (mostly consisting of partial late transcription factor VTFL-1 (ORF045) and envelope phospholipase (ORF011) as well as smaller amounts of partial protein

kinase (ORF130), and DNA-directed RNA polymerase subunit RPO132 (ORF101)), no further analysis was done, and we decided to use an alternative approach.

PCR-free sequencing and sequence analysis

To separate host and virus DNA, methylated DNA was removed from 1 µg of DNA with Microbiome DNA Enrichment kit (NEBNext) according to the manufacturer's instructions. Unmethylated DNA was then subjected to next-generation sequencing (NGS) as described for the first protocol. Before proceeding with the sample, the protocol was tested with 1 µg of cultured reindeer pseudocowpox virus (F00.120R) DNA, from which the entire genome was acquired (assembly performed with Bowtie 2 (*9*) and GQ329669.1 as a reference genome (*10*)).

We were not able to perform a reference-based assembly since no complete genomes of similar viruses are available. Single-end reads were preprocessed and assembled using Lazypipe 2.0 (*11*) with default options and no host filtering. For assembler we used megahit version 1.2.9 (*12*) with the help of in-house scripts.

Sequence alignment, p-distance calculations, and GC-content calculation were performed with MEGA 11 (*13*). Sequences were aligned with Muscle (*14*). A representative of each genus in the subfamily *Chordopoxvirinae* was selected for a phylogenetic analysis of the DNA polymerase gene. Additionally, representatives of each recognized parapoxvirus (PPV) species with complete genome available as well as Molluscum contagiosum virus and Squirrelpox virus as outgroups were selected for phylogenetic analyses of PPVs using the DNA polymerase, early transcription factor, RNA polymerase, and topoisomerase 1 genes. Phylogenetic analyses were performed using amino acid sequences (DNA polymerase) or nucleotide sequences (other genes) using IQ-TREE 2.2.0.3 (*15*) and visualized with iTOL (*16*).

Virus culturing

In an attempt to isolate the virus, the remaining skin biopsy sample (≈10 mg) was homogenized in 500 µl of Dulbecco's phosphate buffered saline + 0.2% bovine serum albumin with a mortar on dry ice. The sample suspension was filtered through 0.45 µm filter (Whatman) to get rid of bacteria. Primary bovine esophagus cells (CCLV, Friedrich-Loeffler-Institute) cultured in Minimum Essential Medium (GIBCO) supplemented with 1% L-glutamine (GIBCO), 1% Non-essential amino acids (GIBCO), 10% sheep serum, 200 IU/ml penicillin (Orion) and 400 µg/ml streptomycin (Sigma) were used in virus culturing. Cells that had been divided on the

previous day were infected with 50 µl of the sample supernatant in a 24-well plate (Nunc). The following passages were infected with 1 ml of the previous passage in a 25-cm^2 cell culture flask (Nunc). Samples were allowed to adsorb for 1 h at 37°C before adding the cell culture medium. Cells were harvested on the seventh day after infection by freezing and thawing three times. The passaging was repeated three times. No cytopathic effect was detected.

References

1. Airas N, Hautaniemi M, Syrjä P, Knuuttila A, Putkuri N, Coulter L, et al. Infection with Possible Novel Parapoxvirus in Horse, Finland, 2013. Emerg Infect Dis. 2016;22:1242–5. PubMed https://doi.org/10.3201/eid2207.151636

2. Inoshima Y, Morooka A, Sentsui H. Detection and diagnosis of parapoxvirus by the polymerase chain reaction. J Virol Methods. 2000;84:201–8. PubMed https://doi.org/10.1016/S0166-0934(99)00144-5

3. Chevreux B, Wetter T, Suhai S. Genome Sequence Assembly Using Trace Signals and Additional Sequence Information. Computer Science and Biology: Proceedings of the German Conference on Bioinformatics (GCB) 1999(9):45–56.

4. Li Y, Meyer H, Zhao H, Damon IK. GC content-based pan-pox universal PCR assays for poxvirus detection. J Clin Microbiol. 2010;48:268–76. PubMed https://doi.org/10.1128/JCM.01697-09

5. Kottaridi C, Nomikou K, Lelli R, Markoulatos P, Mangana O. Laboratory diagnosis of contagious ecthyma: comparison of different PCR protocols with virus isolation in cell culture. J Virol Methods. 2006;134:119–24. PubMed https://doi.org/10.1016/j.jviromet.2005.12.005

6. Meyer H, Pfeffer M, Rziha HJ. Sequence alterations within and downstream of the A-type inclusion protein genes allow differentiation of Orthopoxvirus species by polymerase chain reaction. J Gen Virol. 1994;75:1975–81. PubMed https://doi.org/10.1099/0022-1317-75-8-1975

7. Obon E, Juan-Sallés C, McInnes CJ, Everest DJ. Poxvirus identified in a red squirrel (Sciurus vulgaris) from Spain. Vet Rec. 2011;168:86. PubMed https://doi.org/10.1136/vr.d204

8. Himsworth CG, McInnes CJ, Coulter L, Everest DJ, Hill JE. Characterization of a novel poxvirus in a North American red squirrel (Tamiasciurus hudsonicus). J Wildl Dis. 2013;49:173–9. PubMed https://doi.org/10.7589/2012-02-054

9. Langmead B, Salzberg SL. Fast gapped-read alignment with Bowtie 2. Nat Methods. 2012;9:357–9. PubMed https://doi.org/10.1038/nmeth.1923

10. Hautaniemi M, Ueda N, Tuimala J, Mercer AA, Lahdenperä J, McInnes CJ. The genome of pseudocowpoxvirus: comparison of a reindeer isolate and a reference strain. J Gen Virol. 2010;91:1560–76. PubMed https://doi.org/10.1099/vir.0.018374-0

11. Plyusnin I, Kant R, Jaaskelainen AJ, Sironen T, Holm L, Vapalahti O, et al. Novel NGS pipeline for virus discovery from a wide spectrum of hosts and sample types. Virus Evol. 2020 Jul;6(2):veaa091.

12. Li D, Liu CM, Luo R, Sadakane K, Lam TW. MEGAHIT: an ultra-fast single-node solution for large and complex metagenomics assembly via succinct de Bruijn graph. Bioinformatics. 2015;31:1674–6. PubMed https://doi.org/10.1093/bioinformatics/btv033

13. Tamura K, Stecher G, Kumar S. MEGA11: Molecular Evolutionary Genetics Analysis Version 11. Mol Biol Evol. 2021;38:3022–7. PubMed https://doi.org/10.1093/molbev/msab120

14. Edgar RC. MUSCLE: multiple sequence alignment with high accuracy and high throughput. Nucleic Acids Res. 2004;32:1792–7. PubMed https://doi.org/10.1093/nar/gkh340

15. Minh BQ, Schmidt HA, Chernomor O, Schrempf D, Woodhams MD, von Haeseler A, et al. IQ-TREE 2: New Models and Efficient Methods for Phylogenetic Inference in the Genomic Era. Mol Biol Evol. 2020;37:1530–4. PubMed https://doi.org/10.1093/molbev/msaa015

16. Letunic I, Bork P. Interactive Tree Of Life (iTOL) v5: an online tool for phylogenetic tree display and annotation. Nucleic Acids Res. 2021;49(W1):W293–6. PubMed https://doi.org/10.1093/nar/gkab301

Appendix Table 1: Nucleotide identities as percentages between sequences from the Appendix Figure. Identities within species are indicated with an asterisk.

Species	EqPPV-A5/Horse/Finland/2022	EqPPV-A6/Horse/Finland/2022	EqPPV-A4/Horse/Finland/2022	EqPPV-J2/Horse/Finland/2022	EqPPV-F3/Horse/Finland/2022	EqPPV-F14.1158H/Horse/Finland/2013	JF773701/ORF-F07.821R/Reindeer/Finland/2007	AY386263/ORF-IA82/Sheep/USA/1982	GQ329669/PCPV-F00.120R/Reindeer/Finland/2000	DQ184476/ORF_NZ2/Sheep/New_Zealand/1982	AY386265/BPSV-AR02/Bovine/USA/2002	JF773695/PCPV/F10.3081C/Bovine/Finland/2010	GQ329670/PCPV-VR634/Human/USA/1963	JF773703/ORF-F09.1160S/Sheep/Finland/2009	JF773692/PCPV-F07.798R/Reindeer/Finland/2007	AY453655/Parapoxvirus-DPV/Red_deer/New_Zealand	KM502564/Parapoxvirus-HL953/Red_deer/Germany/2013
EqPPV-A6 (2022)	100*																
EqPPV-A4 (2022)	100*	100*															
EqPPV-J2 (2022)	99*	99*	99*														
EqPPV-F3 (2022)	100*	100*	100*	99*													
EqPPV-F14.1158H (2013)	97*	97*	97*	97*	97*												
JF773701/ORF-F07.821R	84	85	85	84	85	84											
AY386263/ORF-IA82	84	86	86	84	85	84											
GQ329669/PCPV-F00.120R	83	85	85	83	84	85	94	94									
DQ184476/ORF_NZ2	85	87	87	85	86	84	99*	99*	95								
AY386265/BPSV-AR02	82	82	82	82	82	83	88	86	88	87							
JF773695/PCPV/F10.3081C	83	85	85	83	84	85	94	94	99*	94	87						
GQ329670/PCPV-VR634	84	86	86	84	85	86	95	95	99*	96	89	98					
JF773703/ORF-F09.1160S	84	85	85	84	84	83	99*	99*	94	99*	86	93	95				
JF773692/PCPV-F07.798R	83	85	85	83	84	85	94	94	100*	95	88	100*	99*	94			
AY453655/Parapoxvirus-DPV	80	81	81	79	80	82	83	84	87	85	85	87	87	84	87		
KM502564/Parapoxvirus-HL953	80	82	82	79	81	81	83	85	87	85	86	87	88	85	87	100*	
KY382358/Parapoxvirus-AFK76s1	82	83	83	81	82	82	80	80	82	81	80	82	84	80	83	80	80

Appendix Table 2: Primers used in the first sequencing attempt.

Primer name	Sequence	Target	Ref
PPP1	GTCGTCCACGATGAGCAGCT	Major envelope protein of parapoxviruses/ envelope phospholipase (ORF011)	(*2*)
PPP4	TACGTGGGAAGCGCCTCGCT		
Pan-pox high GC For	CATCCCCAAGGAGACCAACGAG	RNA polymerase of poxviruses with >60% GC-content (ORF056)	(*4*)
Pan-pox high GC Rev	TCCTCGTCGCCGTCGAAGTC		
Parapox 045F	CCTACTTCTCGGAGTTCAGC	Late transcription factor 1 of Orf virus (ORF045)	(*5*)
Parapox 045R	GCAGCACTTCTCCTCGTAG		
ATI up1	AATACAAGGAGGATCT	A-type inclusion body protein gene of orthopoxviruses (ORF103–104)	(*6*)
ATI low1	CTTAACTTTTTCTTTCTC		
Pan-pox low GC For	ACACCAAAAACTCATATAACTTCT	Metalloprotease gene of orthopoxviruses (ORF037)	(*4*)
Pan-pox low GC Rev	CCTATTTTACTCCTTAGTAAATGAT		
A3LFor1	CNTCHACNMABRAYTGG	P4b precursor gene of chordopoxviruses (ORF079)*	(*7*)
A3LRev3	TGYTCYTCRTCNGHCAT		
F10LF958	GAYYTNAARCCNGAYAA	Protein kinase gene of chordopoxviruses (ORF130)*	(*8*)
F10LR1167	AARTGRAARTCRTARWACCA		

*Degenerate primers, which have been used to successfully amplify PCR products from distinct squirrel poxviruses

Appendix Table 3: Amino acid identities (as percentages) of the DNA polymerase gene between F14.1158H, parapoxviruses, and representatives of other chordopoxviruses. Identities within genus are indicated with an asterisk.

Virus	SPPV	SWPV	CRV	FWPV	YMTV	SQPV	DPV	MYXV	EMCLV	MOCV	CPXV	VACV	MPXV	PCPV/ F00.120R	PCPV/ VR634	GSEPV	RDPV	ORF/ NZ2	ORF/ HSN-20	BPSV
MZ682626/SWPV/Suipoxvirus	75																			
NC_008030/CRV/Crocodylidpoxvirus	45	45																		
NC_002188/FWPV/Avipoxvirus	53	54	47																	
NC_005179YMTV/Yatapoxvirus	70	68	45	52																
NC_022563SQPV/Sciuripoxvirus	58	57	50	50	59															
NC_006966/DPV/Cervidpoxvirus	76	78	45	53	71	60														
NC_001132/MYXV/Leporipoxvirus	75	76	46	52	70	59	77													
MN339351/EMCLV/Molluscipoxvirus	51	52	53	51	51	59	53	52												
MH320556/MOCV/Molluscipoxvirus	51	52	52	52	50	59	53	51	73*											
NC_003663/CPXV/Brighton_Red/Orthopoxvirus	66	66	47	51	67	61	68	67	54	54										
NC_006998/VACV/West_Reserve/Orthopoxvirus	66	66	48	51	67	61	68	67	54	53	99*									
ON959143/MPXV/MPX-96/Orthopoxvirus	66	65	47	51	66	60	67	66	54	53	98*	98*								
GQ329669/PCPV/F00.120R/Parapoxvirus	54	54	48	48	53	59	54	55	55	53	57	57	56							
GQ329670/PCPV/VR634/Parapoxvirus	54	54	49	48	54	60	54	54	55	53	56	57	56	98*						
KY382358/GSEPV/Parapoxvirus	55	54	48	47	54	58	55	54	54	52	56	56	56	84*	84*					
KM502564/RDPV/Parapoxvirus	53	52	48	46	52	58	54	53	55	53	56	56	55	85*	86*	85*				
DQ184476/ORF/NZ2/Parapoxvirus	54	54	49	47	54	59	54	55	55	53	57	57	56	95*	94*	84*	86*			
MW537048/ORF/HSN-20/Parapoxvirus	54	54	48	47	54	59	54	55	55	53	57	57	56	95*	94*	85*	86*	99*		
NC_005337/BPSV/Parapoxvirus	54	54	48	48	54	59	54	55	54	53	57	57	56	88*	88*	85*	87*	87*	87*	
F14.1158H	53	53	50	46	52	60	54	53	56	54	57	57	56	79*	80*	76*	78*	79*	79*	78*

CRV = Crocodilepox virus, EMCLV = Equine molluscum contagiousum-like virus, MOCV = Molluscum contagiousum virus, SQPV = Squirrelpox virus, CPXV = Cowpox virus, MPXV = Monkeypox virus, VACV = Vaccinia virus, YMTV = Yaba monkey tumor virus, SPPV = Sheeppox virus, MYXV = Myxoma virus, SWPV = Swinepox virus, DPV = Deerpox virus, FWPV = Fowlpox virus

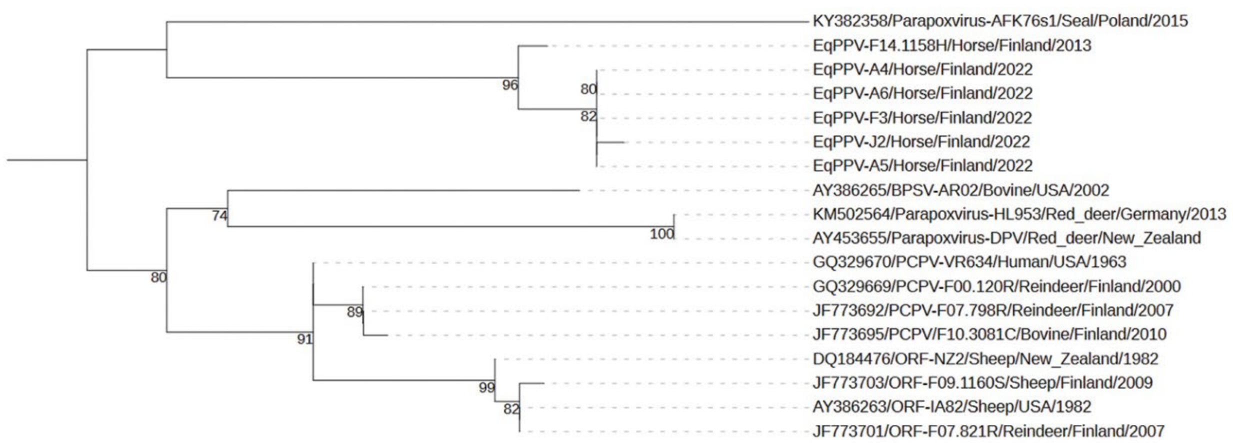

Appendix Figure: Phylogenetic tree of partial envelope phospholipase gene (ORF011, bp 449–620) including F14.1158, 5 sequences from 2022 pastern dermatitis outbreak, and selected representatives of other parapoxvirus species. Bootstrap values above 70% are shown next to the nodes.
